# Supplementary figures and images for: An ex vivo rat trachea model reveals abnormal airway physiology and a gland secretion defect in cystic fibrosis
Source: PLoS One. 2023 Oct 24;18(10):e0293367. doi: 10.1371/journal.pone.0293367 (PMC10597513; doi:10.1371/journal.pone.0293367)

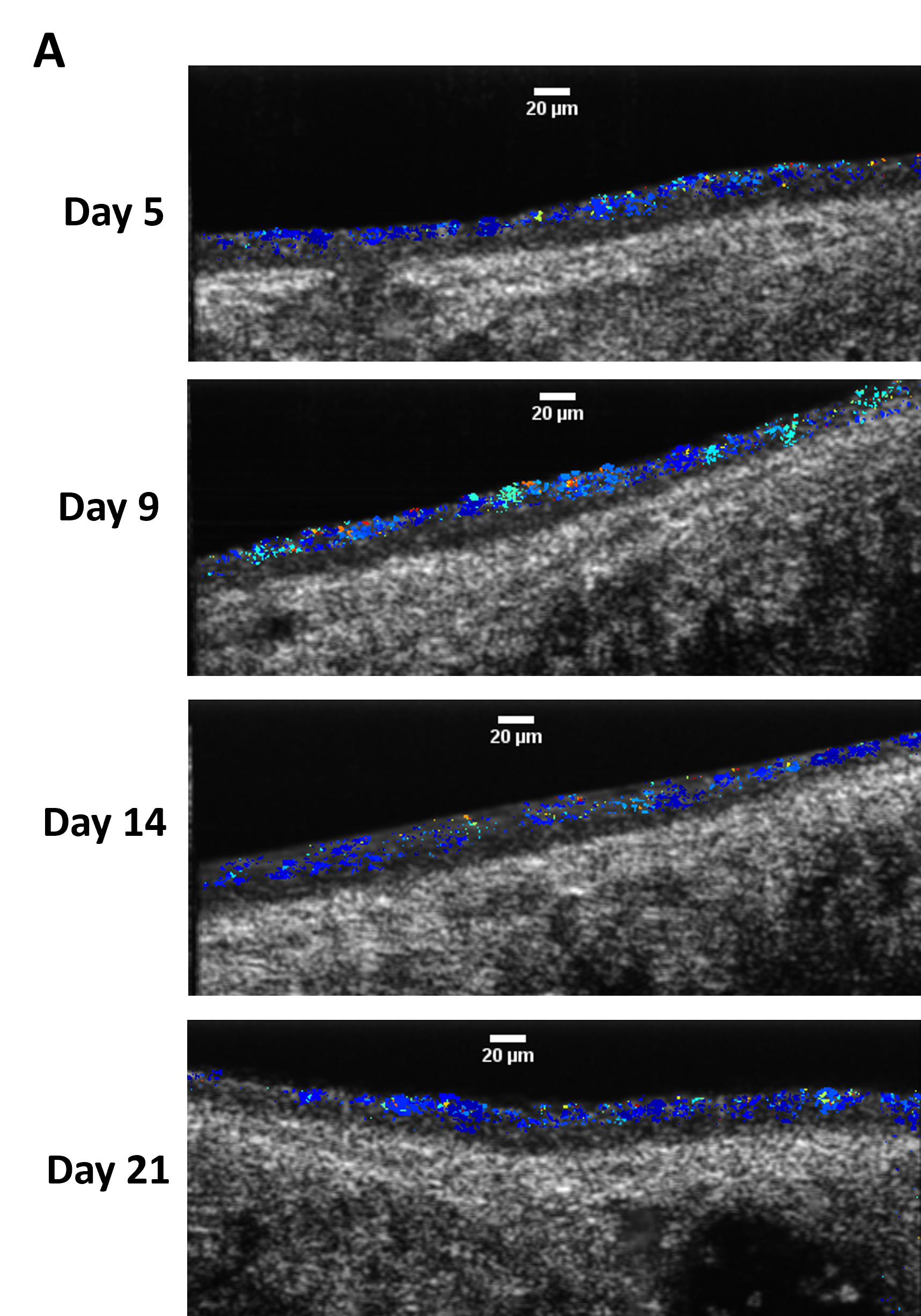

Supplement: S1 Fig — (A) Representative μOCT images with color map of cilia coverage on an ex vivo trachea at 5, 9, 14, and 21 days after explanting. Scale bars are 20 μm. μOCT- micro-Optical Coherence Tomography. (TIF) [file pone.0293367.s001.tif]

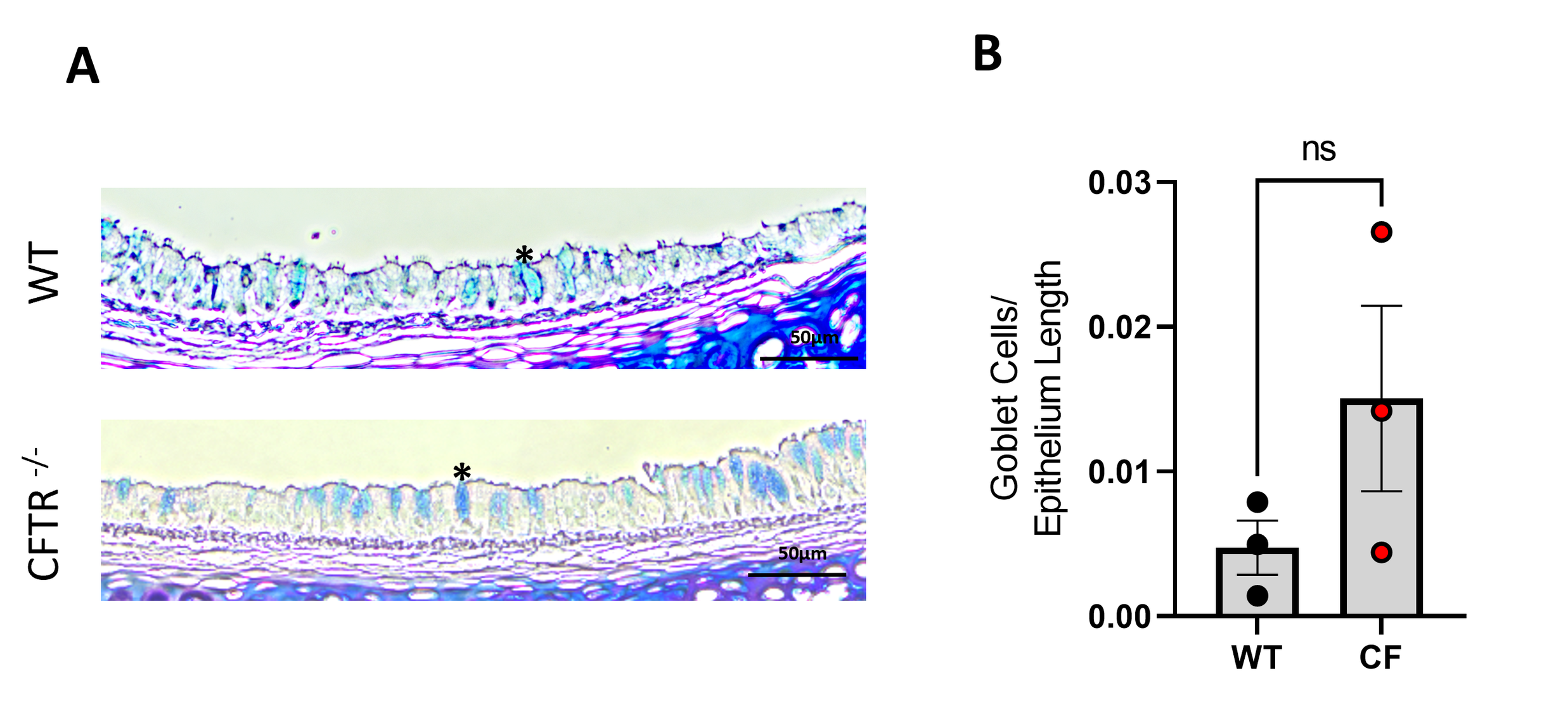

Supplement: S2 Fig — (A) Representative AB-PAS stained tissue of WT and CFTR-/- ex vivo tracheae 7 days after explanting, depicting goblet cells (*). (B) Quantification of goblet cells per mm of tracheal epithelium. N = 3/condition. nsP>0.05 by unpaired t-test. Scale bars are 50 μm. AB-PAS- alcian blue-periodic acid Schiff’s. (TIF) [file pone.0293367.s002.tif]

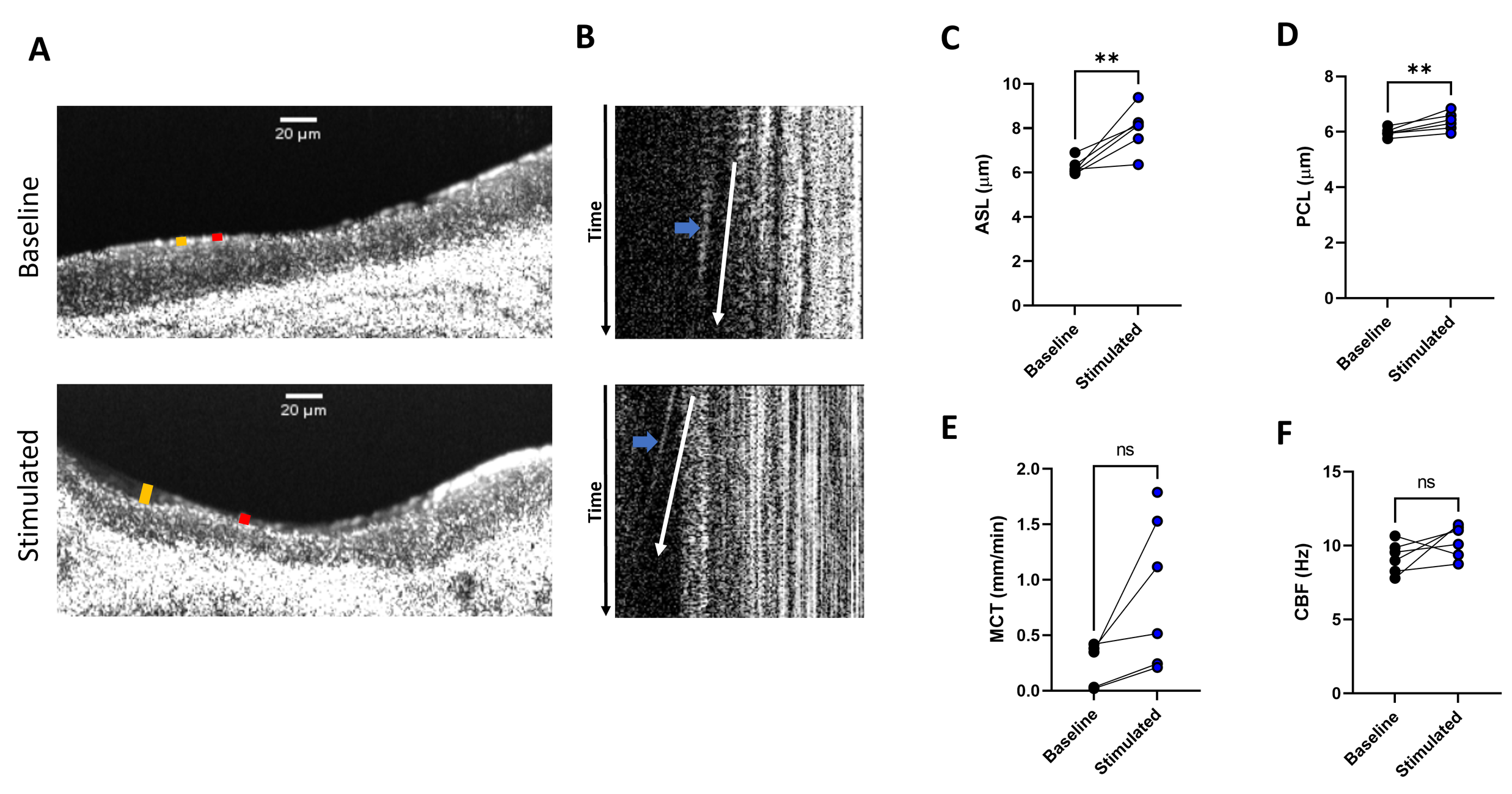

Supplement: S3 Fig — (A) Representative μOCT images of WT ex vivo tracheae at baseline and after stimulation with 10 μM carbachol for 1 hour, depicting the ASL (orange bar) and PCL (red bar). (B) Time-dependent reprocessed images show tracks of mucus particles above the epithelial surface of WT ex vivo tracheae at baseline and after stimulation with carbachol; the more horizontal direction of particle streaks (blue arrow) indicates more rapid transport. (C-F) Regions of interest were measured and averaged for each trachea for (C) ASL, (D) PCL, (E) MCT, and (F) CBF. N = 5-7/condition. nsP>0.05, **P<0.01 by paired t-test. Scale bars are 20 μm. μOCT- micro-Optical Coherence Tomography, ASL- airway surface liquid, PCL- periciliary liquid, MCT- mucociliary transport, CBF- ciliary beating frequency. (TIF) [file pone.0293367.s003.tif]
