# Supplementary material for: An ex vivo rat trachea model reveals abnormal airway physiology and a gland secretion defect in cystic fibrosis
Source: PLoS One. 2023 Oct 24;18(10):e0293367. doi: 10.1371/journal.pone.0293367 (PMC10597513; doi:10.1371/journal.pone.0293367)
Supplement: S1 Raw images — (PDF) [file pone.0293367.s014.pdf]

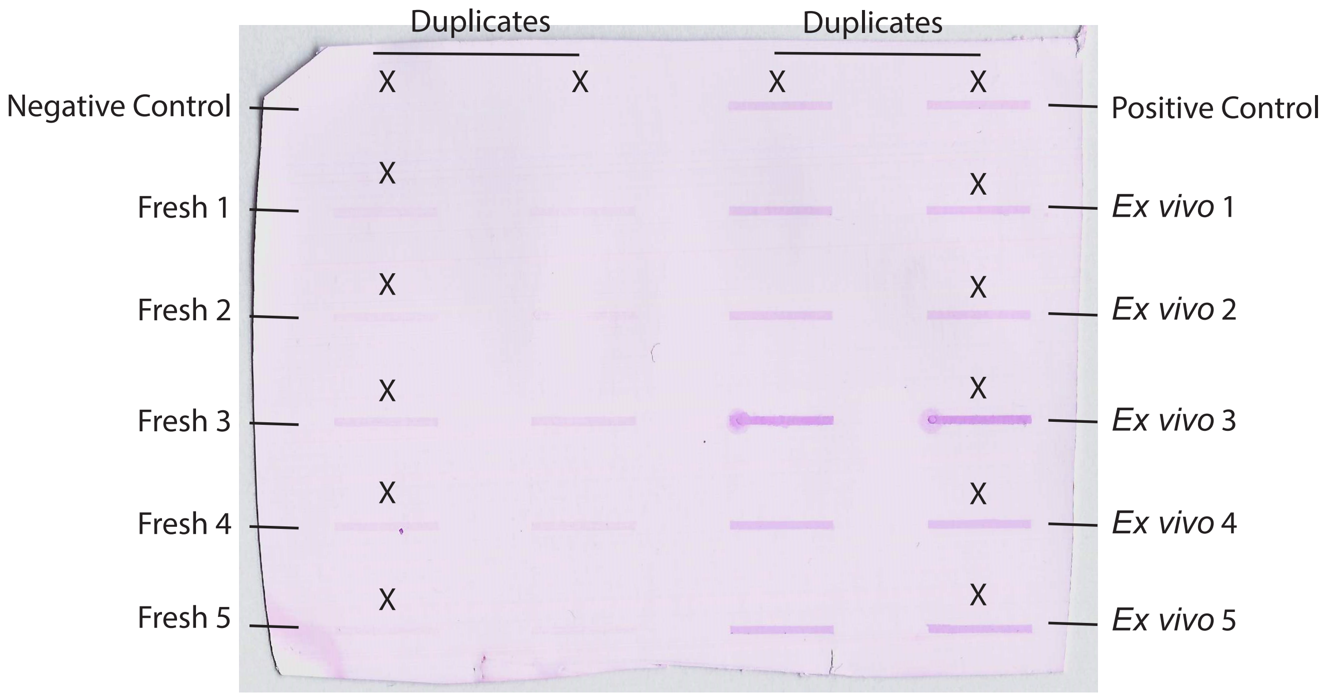

Raw blot image of Figure 6 panel D. Image was captured in TIFF file format using a HP color computer scanner. Lanes annotated with "fresh" indicate that the blotted samples were mucus collections from freshly excised tracheae. Lanes annotated with "Ex *vivo*" indicate that the blotted samples were mucus collections from ex *vivo* tracheae cultures. Ascending numbers (1-5) indicate 5 different tracheae from which mucus was collected and blotted for each group.
